# Role of Epigenetics in Stem Cell Proliferation and Differentiation: Implications for Treating Neurodegenerative Diseases

**DOI:** 10.3390/ijms17020199

**Published:** 2016-02-02

**Authors:** Bhairavi Srinageshwar, Panchanan Maiti, Gary L. Dunbar, Julien Rossignol

**Affiliations:** 1 Field Neurosciences Institute laboratory for Restorative Neurology at Central Michigan University, Mt. Pleasant, MI 48859, USA; srina1b@cmich.edu (B.S.); maiti1p@cmich.edu (P.M.); dunba1g@cmich.edu (G.L.D.); 2 Program in Neuroscience, Central Michigan University, Mt. Pleasant, MI 48859, USA; 3 Department of Psychology, Central Michigan University, Mt. Pleasant, MI 48859, USA; 4 Field Neurosciences Institute, St. Mary’s of Michigan, Saginaw, MI 48604, USA; 5 College of Medicine, Central Michigan University, Mt. Pleasant, MI 48859, USA

**Keywords:** epigenetics, histone modifications, stem cells, neurodegenerative diseases, Huntington’s disease, Alzheimer’s disease, mesenchymal stem cells (MSCs), neural stem cells (NSCs), induced pluripotent stem cells (iPSCs)

## Abstract

The main objectives of this review are to survey the current literature on the role of epigenetics in determining the fate of stem cells and to assess how this information can be used to enhance the treatment strategies for some neurodegenerative disorders, like Huntington’s disease, Parkinson’s disease and Alzheimer’s disease. Some of these epigenetic mechanisms include DNA methylation and histone modifications, which have a direct impact on the way that genes are expressed in stem cells and how they drive these cells into a mature lineage. Understanding how the stem cells are behaving and giving rise to mature cells can be used to inform researchers on effective ways to design stem cell-based treatments. In this review article, the way in which the basic understanding of how manipulating this process can be utilized to treat certain neurological diseases will be presented. Different genetic factors and their epigenetic changes during reprogramming of stem cells into induced pluripotent stem cells (iPSCs) have significant potential for enhancing the efficacy of cell replacement therapies.

## 1. Introduction

All cells in an organism are derived from pre-existing cells, beginning with the fertilized egg, which forms the blastocyst, which, in turn, gives rise to the cells of the entire organism [1]. These stem cells have the unique property of being totipotent, meaning they can give rise to any type of cell in the organism, including a placenta. The two major properties of stem cells are proliferation and differentiation. Stem cells can proliferate and differentiate into appropriate lineages to form specialized cells. The potential for each type of stem cell to become specialized depends on its plasticity, or the degree to which it is, or can become, totipotent, pluripotent (able to become any cell other than placenta) or multipotent (able to become many, but not all cell types) [2]. Owing to their plasticity, stem cells are very useful in the field of regenerative medicine, especially as a potential cell-replacement therapy for many diseases. For example, the bone marrow-derived mesenchymal stem cells have the potential to secrete brain-derived neurotrophic factor (BDNF), vascular endothelial growth factor (VEGF), nerve growth factor (NGF) and insulin-like growth factor 1 (IGF-1) as a therapy for neurodegenerative disorders and spinal cord injury [3,4,5]. Within the context of treatments for neurodegenerative disorders, stem cells have enormous therapeutic potential, particularly: (1) mesenchymal stem cells (MSCs), which can be derived from bone marrow (BM-MSCs); (2) neural stem cells (NSCs), derived from embryonic mouse brain tissues (eNSCs); and (3) induced pluripotent stem cells (iPSCs). In particular, the iPSCs can be derived from fibroblasts and then driven into a neuronal lineage, differentiating into what appears to be mature neurons, which may be able to replace lost nerve cells. An added advantage of using exogenous stem cells for treatment strategies is that they can be genetically modified to overexpress certain proteins that become downregulated in certain brain diseases [6,7,8]. As discussed above, the MSCs can be genetically modified to overexpress brain-derived neurotrophic factor (BDNF), which is one of the major trophic factors that is downregulated in Huntington’s disease (HD), a neurodegenerative disorder characterized by loss of medium spiny neurons in the striatum [7]. Similarly, MSCs can be altered to express glial-derived neurotrophic factor (GDNF) to promote dopaminergic neuronal sprouting to treat Parkinson’s disease (PD). In addition, these MSCs can also be used to overexpress nerve growth factor (NGF) to alleviate memory deficits in Alzheimer’s disease (AD) [9]. However, one of the drawbacks of stem cell transplantation is the immune response that arises during allogenic transplantation [10]. However, at least one study has demonstrated that adult stem cells, when used in their undifferentiated form, can escape immune rejection and do not pose any adverse effects [11].

Given the relative ease of manipulation, exogenously-administered stem cells can be differentiated into a particular cell lineage to secrete specific proteins. However, the precise mechanism(s) whereby stem cells normally proliferate and differentiate into specific lineages, or even what determines the fate of these stem cells, is not yet understood. Gaining new insights into these endogenous processes may assist researchers with how exogenous stem cells may be more efficiently manipulated in ways that would optimize their therapeutic efficacy for treating neurodegenerative diseases.

Manipulation of stem cells involves epigenetic regulation, which can be used to drive these stem cells towards the lineage of interest, preparing them for subsequent use as a cell replacement therapy [12]. Several recent studies have indicated that epigenetic processes play an essential role in normal gene expression and cell differentiation [13,14]. Understanding the role of these epigenetic processes should help successful stem cell reprogramming in the creation of iPSCs, which require several genetic factors [15]. In culture, some of the stem cells undergo epigenetic changes, while others remain unchanged during passaging or reprogramming. Knowing why certain cells utilize epigenetic processes during differentiation, both *in vitro* and *in vivo*, may help us devise new ways in which the epigenetic process can be used to enhance or control proliferation and differentiation of stem cells. In this context, our current understanding of the molecular mechanisms involved in epigenetic control of stem cell differentiation into various cell lineages, with special attention as to how these mechanisms can be leveraged as therapeutic tools for various neurological diseases, will be reviewed.

## 2. Epigenetics Regulate Cell Differentiation

**Figure 1:**
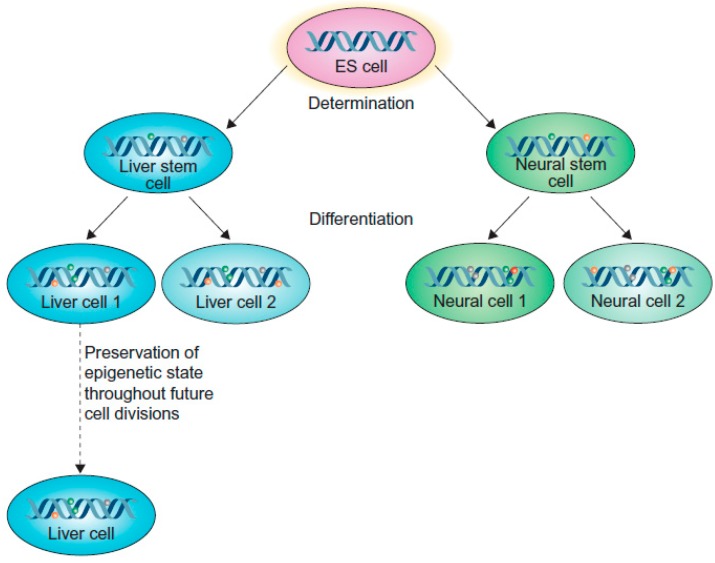
Epigenetic memory at the cellular level. Epigenetic memory is maintained during differentiation of neurons and liver cells from the precursor embryonic cells (adopted from [19] Copyright 2013 with permission from Elsevier).

It is strongly believed that there are some signals at the epigenetic level that regulate the fate of the stem cells [14,16]. Though all of the cells in our body contain the same genetic makeup, these genes are not necessarily active at all times, rather they are expressed at times when needed, in a highly controlled fashion. This tightly-regulated gene expression in our body is governed by epigenetics. The mechanism of gene regulation does not depend on the DNA sequences [17], but is perpetuated as a “memory” that is carried on from one cell to another during cell division [18]. For example, liver stem cells and neuronal stem cells are derived from the same precursor cells. Obviously, their genetic makeup is expected to be the same, but during differentiation, they are able to form into either mature liver cells or neurons. As illustrated in Figure 1, a particular fate of a cell is determined by their specific pattern of gene expression [19].

Hence, the cellular memory is retained in these stem cells and pushes them to become either neurons or liver cells, rather than any other type of cell. However, the classical way of explaining “epigenetics” has been revised, due to the understanding that the epigenetic mechanisms of cellular “memory inheritance” are somewhat different in neurons, given their inability to divide beyond their initial post-mitotic divisions [19,20]. For unknown reasons, the epigenetic mechanisms and memory recall have become permanently entrenched in neurons, preventing their proliferation. Hence, cell division is no longer thought to be a requisite for the transfer of epigenetic signals, as exemplified by non-dividing neurons [19].

**Figure 2:**
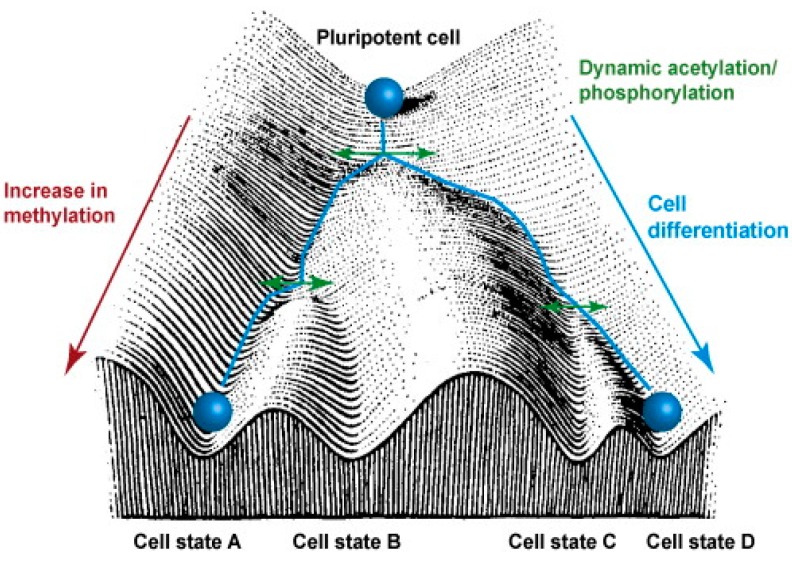
Waddington’s epigenetic landscape. This image provides an analogy of how the pluripotent stem cells give rise to specialized cells as they undergo epigenetic changes, such as methylation, acetylation and phosphorylation (adopted from [23] Copyright 2010 with permission from Elsevier).

The epigenetic analysis of the stem cell fate dates back to early 1900s, when biologist Conrad Hal Waddington proposed the “epigenetic landscape”, an image that depicts how various epigenetic mechanisms determine what these stem cells become in the end. Waddington’s landscape consists of troughs and crests (or ridges and valleys) that branch out downhill through which the pluripotent cells roll down. The surface of the landscape represents the genes that have undergone epigenetic modifications [21,22] (see Figure 2).

As the pluripotent cell rolls down “the differentiation hill”, the path it takes depends on the conformation of the surface, which represents the presence of various epigenetically-modified genes that the cell encounters in its downward path, which ultimately determines the fate of the cell [22,24]. In reality, the epigenetic modifications that the pluripotent cells experience during their proliferation determine what type of specialized cell will be created.

The major molecules involved in regulating gene expressions are the histones or the DNA-binding proteins that undergo various modifications, such as methylation, acetylation, ubiquitination and phosphorylation [25] (Table 1). In short, DNA methylation involves methylation of cytosine at the 5’ position to give rise to 5’ methyl-cytosine, which is mediated by the enzyme DNA methyltransferases (DNMT). Usually, DNA methylation is associated with the silencing of genes, but there are some exceptions to this rule. Methylation of histone 3 at lysine at position 4 (H3K4me3) activates the gene, whereas the lysine at position 27 (H3K27me3) silences the gene [26]. Similarly, histone acetylation involves adding an acetyl group to the lysine residue at the N-terminal of the histone. This reaction is regulated by the histone acetyltransferase (HAT) and histone deacetylase (HDAC) enzymes. These two enzymes have opposing actions on each other [27]. HDACs remove an acetyl group from the lysine amino acid residue, maintaining a positive charge on the amino acid, whereas HATs regulate the transfer of acetyl group, neutralizing the charge on the lysine. With acetylation and deacetylation occurring at various sites on the histone, the stability maintained by the electrostatic interaction is disrupted, thereby regulating the gene expression. [27,28]. Histone phosphorylation involves the addition of phosphate groups to the amino acids, such as serines, threonines and tyrosines. The histone phosphorylation is catalyzed by kinase and phosphatase enzymes, thereby adding and removing phosphate to and from the amino acids, respectively [29]. Similar to the histone acetylation process, gene regulation by histone phosphorylation involves modifying the charge on the amino acid. For example, the kinase enzyme adds a phosphate to the amino acid side chain, making the histone negatively charged and modifying the chromatin structure [27].

**Table 1:** Epigenetic regulations of gene expression by histones. Summary of the type of modifications that histones undergo to regulate gene expression by repressing or enhancing gene transcription, thereby silencing and activating them, respectively.

Molecules Regulate Gene Expression	Types of Modification	Examples	Outcomes
Histones	Methylation [30]	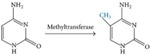	Histone methylation commonly silences the gene by repressing transcription, involving factors such as H3K27me3 [26].
	Acetylation [31,32]	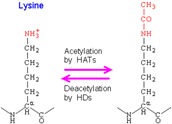	Histone acetylation activates genes by enhancing the transcription, involving factors such as H3K4ac [31].
	Phosphorylation [33,34]	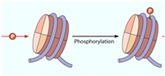	Histone phosphorylation helps in chromatin remodeling and repairs the damaged DNA, such as H3T6 phosphorylation [35].

The other type of molecules involved in epigenetic modifications are micro-RNAs (miRNA). These miRNAs are small non-coding RNA molecules that are involved in gene silencing at the post transcriptional level. They play a major role in tissue-specific gene expression during development [36].

The gene expression regulation by miRNAs is briefly discussed in Table 2.

**Table 2:** Epigenetic regulations of gene expression by micro-RNAs. Description of the effects of methylated mRNAs that are associated with gene repression.

Molecules that Regulate Gene Expression	Types of Modification	Examples	Outcome
Micro-RNA	Methylation	miR9	Associated with cancer metastasis by gene repression [37].
		miR-34b/c	Affects the gene expression of miR-34: miR-34a, miR-34b and miR-34c; and it is associated with colorectal cancer [38].
		miR-124	Associated with brain tumor by transcription repression [39].

Apart from histones, there are other types of DNA binding proteins, such as polycomb group proteins (PcG), heterochromatin protein (HP1) and DNA-binding zinc finger protein (ZnFn), that regulate gene expression. These proteins are very specific to a particular DNA sequence on the genome and modify the chromatin structure to regulate gene expression [40] (Table 3).

**Table 3:** Epigenetic regulations of gene expression by DNA binding proteins. Summary of the gene expression regulations by methylated DNA binding proteins, such as polycomb group proteins, heterochromatin proteins and DNA binding zinc finger proteins.

Molecules Regulate Gene Expression	Type of Modification	Examples	Outcomes
DNA-binding proteins	Methylation	Polycomb group proteins (PcG)	Play a role in cellular differentiation by repressing transcription [41].
		Heterochromatin protein (HP1)	Include many functions, like repressing genes by heterochromatin formation, regulates binding of complexes to centromere and maintains chromatin integrity [42].
		DNA binding zinc finger protein (ZnF)	Regulates transcription processes, such as C2H2 ZnFs [43].

These epigenetic modifications play a role in stem cell proliferation and differentiation, which will be discussed in detail in the upcoming sections of this review. Interestingly, exercise and diet can also have an impact on epigenetic modification and gene expression. During exercise, the body releases exosomes containing miRNA, which plays a role in gene expression. Similarly, intake of vitamins (B6 and B12) has an impact on the homocysteine, which is a by-product obtained from DNA/RNA methylation. However, the roles of diet and exercise are beyond the focus of this review [44].

As mentioned earlier, the differentiation of MSCs, NSCs and iPSCs into neuronal lineages constitutes a major interest for researchers who are searching for potential therapies for neurodegenerative disorders.

Hence, epigenetic mechanisms have a role in determining how successfully the MSCs, NSCs and iPSCs can differentiate into neuronal and glial lineages and to what extent they are able to exert therapeutic effects for treating various neurodegenerative disorders.

## 3. Epigenetic Mechanisms in Mesenchymal Stem Cells and Huntington’s Disease

Huntington’s disease is an autosomal dominant, late-onset neurodegenerative disease characterized by the degeneration of medium spiny neurons in the striatum of the brain [7]. The main cause of the disease is the CAG repeat expansion on the Huntingtin gene (*HTT*), giving rise to abnormal huntingtin protein characterized by an extended polyglutamine tract (poly Q). This leads to intracellular aggregation of the abnormal protein, which then becomes toxic to the neurons [45,46]. Another characteristic observed in HD is a downregulation of trophic factors. Use of MSCs, which can be engineered to overexpress these factors, may offer a potential treatment for HD. Use of MSCs for clinical applications has enormous potential in the field of neuroscience. MSCs can maintain tissue homeostasis, have very low immune responses and are abundantly present in the bone marrow (BM) [47]. During the last few years, our lab has focused considerable effort on modifying BM-MSCs to secrete increased levels of neurotrophic factors. When these modified BM-MSCs are transplanted into animal models of HD, they create a more suitable environment in the brain by reducing inflammation and restoring lost neurotrophic support to help compensate the neurons that are lost through the disease process. Previous work conducted by the present authors has indicated that MSCs, which have been modified to overexpress BDNF, which, in turn, is downregulated in the brains of the HD patients, can ameliorate deficits in a rodent model of HD [7]. Since BDNF is required for maintaining healthy neurons, MSCs overexpressing BDNF can compensate for the deficient levels in HD brains, thereby reducing the number of lost neurons in the degenerated regions of the HD brain [9].

Given that BM-derived MSCs are multipotent and can differentiate into three lineages (osteogenic, adipogenic and chondrogenic), depending on the molecular mechanisms of the genes [48], these cells can provide a useful tool for studying the epigenetic processes in cellular differentiation. Osteopontin (OPN), peroxisome proliferator-activated receptors gamma 2 (PPAR-γ2) and fatty acid binding protein 4 (FABP4) are the genes associated with osteogenic and adipogenic pathways [49]. To push these MSCs away from their traditional lineages into a neuronal lineage, they must be forced to undergo epigenetic changes. This is achieved by expanding them in culture or passaging them, during which time some of the genes of these cells undergo methylation and acetylation [50]. As these MSCs expand in culture, the epigenetic status of OPN, PPAR-γ2 and FABP4 undergoes changes, and the fate of the cells becomes different, as they are driven towards a neuronal lineage. Using the analogy of Waddington’s epigenetic landscape, during cell passaging, these MSCs take a different route on the surface of the landscape. Specifically, the levels of the marker, H3K9Ac (acetylation of lysine at the ninth position on histone 3, which is associated with gene activation), are decreased in the promotor regions of OPN, PPAR-γ2 and FABP4, suggesting that these genes are less likely to become activated [51]. In such a scenario, these MSCs are less likely to attain a bone or bone-marrow lineage, allowing for a shift towards a neuronal lineage.

Since passaging these MSCs has an impact on determining their fates, work in the lab of the present authors has been conducted to assess the effects of the number of passages (three to eight *versus* 40 to 50 passages) on the characteristics and efficacy of BM-MSCs. Both groups of passaged cells were then transplanted (allotransplants) into the R6/2 mouse model of HD. The outcomes of this study demonstrated that the BM-MSCs with a higher number of passages in the brain were more effective in reducing the behavioral deficits observed in this mouse model of HD [52]. This indicates that passaging the BM-MSC for 40 to 50 times induced them to generate a sub-population of cells that created an environment that produced more trophic factors, like BDNF. This may have created a more suitable microenvironment for the host cells to function more effectively than did the MSCs that were passaged only three to eight times.

Teven and colleagues in 2011 [53] showed that the role of H3K27me3 (methylation of lysine at the 27th position on histone 3) is associated with gene repression in the thyroid hormone receptor interactor-10 gene (Trip10) promoter, *in vitro*. This leads to methylation of MSCs and reduces the expression of the Trip10 gene, thereby directing the MSCs to take on osteogenic and neuronal lineages.

In addition to these epigenetic changes, there are various other epigenetic regulators and markers that occur in various genes of MSCs that determine the cell fate. These are discussed in detail elsewhere [54].

## 4. Epigenetic Mechanism in Adult Neural Stem Cells and Alzheimer’s Disease

Alzheimer’s disease is a neurodegenerative disorder characterized by the accumulation of hyperphosphorylated tau, which forms neurofibrillary tangles (NFTs) in the intracellular spaces, and amyloid beta protein (Aβ), which constitutes the amyloid-plaques in extracellular spaces [55,56]. The major candidate genes involved in AD are Presenilin 1 and 2 (PS1 and PS2) and amyloid precursor protein (APP) [56,57]. Differentiated neural stem cells could be used as a potential treatment for AD. Neural stem cells (NSCs) are precursors for the mature neurons and are present in niche microenvironment areas of the brain [58]. The majority of the NSCs in the brain are present in the sub-ventricular zone (SVZ) and the sub-granular zone (SGZ). The NSC niche areas in the central and the peripheral nervous systems are different from each other, but the signals from these niches coordinate to form the final fate of the NSCs. The NSC population in the niche and its differentiation are regulated by genetic factors, such as growth and transcription factors, as well as environmental factors, such as stress, depression and anxiety [59]. These NSCs undergo epigenetic changes that control both the intrinsic and extrinsic signals, before they become specialized. The three types of epigenetic changes associated with NSCs are DNA methylation, histone modifications and miRNAs. These epigenetic changes interact and depend on each other to push the NSCs into mature neurons and glial cells [60].

### 4.1. DNA Methylation

Mature neurons are usually derived from neurospheres, which are maintained in the presence of growth factors (epidermal and fibroblast growth factors). This ensures that the neurospheres proliferate and do not differentiate, but once the neurospheres enter a different environment that does not have these growth factors, they start to differentiate, producing mature neurons and glial cells from their progenitors. These supplements, which alter the environment of the neurospheres, help to determine their fate [61]. It was discovered that the NSCs in the neurospheres express DNA methyltransferase enzyme, confirming that these NSCs, in their undifferentiated form, undergo DNA methylation. The absence of growth factors triggers epigenetic mechanisms, which reduce the amount of DNMT and DNA methylation [62]. Reduction in DNA methylation leads to the activation of the genes that are necessary for the induction of differentiation and transformation of NSCs into neurons.

### 4.2. Histone Modifications

Adult neurogenesis involves histone acetylation and histone methylation of the genes that are mediated by histone acetyl transferase, deacetylase and histone demethylase enzymes, respectively [63]. Histone deacetylation by HDAC is essential for these cells to undergo neurogenesis, for both neuronal and glial lineages [64]. Inhibition of deacetylase enzyme leads to reduced proliferation of cells, and the “stemness” of the glial cells is lost, resulting in a lesser number of oligodendrocytes and astrocytes that can be formed. Interestingly, it has been shown that the neuronal lineage was not affected by this mechanism [65]. In a similar way, there is other evidence that suggests that histone-3 acetylation leads to increased neuronal cell fate and decreased glial cell fate [66]. This could be induced by using valproic acid, which is a well-known inhibitor of the HDAC enzyme [13]. The environment, particularly the type of supplementations that are available for the neurospheres or the NSCs, can play a vital role in determining the path taken in Waddington’s landscape.

**Figure 3:**
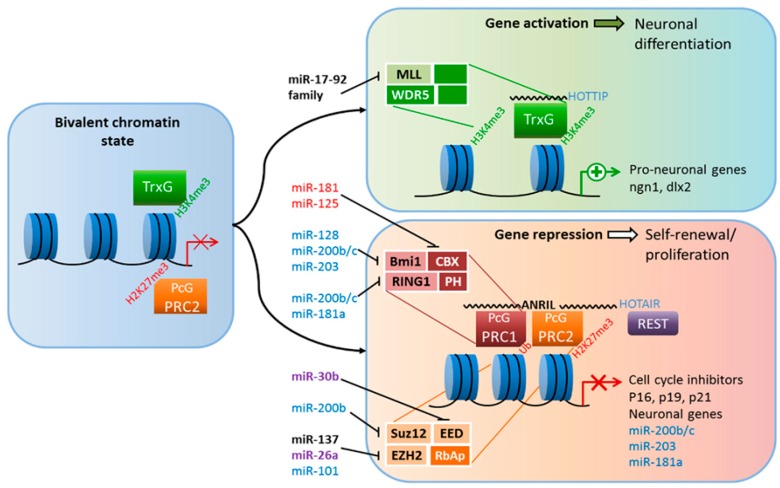
Role of the polycomb group protein complex and the trithorax protein complex in neurogenesis. The trithorax complex (TrxG) is associated with the activation of histone, leading to neuronal differentiation, while the polycomb complex (PcG) is associated with repressive histones, leading to decreased proliferation (adopted from [68]).

DNA methylation, again, has a prominent role in neurogenesis [13]. Two antagonistic complexes modulate the epigenetic regulation of NSCs: the polycomb group of protein complex (PcG) and the trithorax protein complex (TrxG). Previous studies have looked at these complexes in detail, as discussed below. These complexes lead to gene repression and activation by associating with H3K27me3 and H3K4me3, respectively. The TrxG, member of mixed-lineage leukemia-1 (Mll-1), is associated with the activated histone, H3K4me3, which activates the genes required for the NSC differentiation. On the other hand, the PcG consists of the B-lymphoma Mo-MLV insertion region-1 homolog (BMI1-1), which is associated with the repressive histone H3K27me3 (Table 4). Therefore, increase in the repressive histones elevate levels of BMI-1, resulting in decreased NSC proliferation (Figure 3). In order to rescue neurogenesis caused by repressive histones, DNA methyltransferase-3a is vital. This enzyme is an antagonist to PcG and the repressive histone, H3K27me3, allowing for the activation of histone H3K4me3, which can lead to successful neurogenesis [13,60,67].

### 4.3. Micro-RNAs

Micro-RNAs constitute a small, non-coding RNA of about 25 nucleotides in size that can inhibit the expression of developmental genes at the post-transcriptional level. There are about 2000 different types of miRNAs found in the human brain [69]. These miRNAs, in particular, miRNA-9, along with Tlx (orphan nuclear receptor homologue of the Drosophila tailless gene), form a feedback mechanism and regulates neurogenesis. Similarly, the miRNA, Let b, interacts with Tlx and cyclinD and induces NSC differentiation by suppressing proliferation [13,70]. Another interesting miRNA, which is associated with neurogenesis, is miRNA-124. This miRNA is very specific to the brain and plays a major role in controlling the rate at which the neurogenesis takes place. These miRNAs interact with JAG1, SOX9 and DLX2 genes present in the SVZ and promote neural progenitor renewal, glial cell renewal and the production of inter-neurons, respectively [13,71] (see Table 4).

**Table 4:** Epigenetic regulations of gene expression by micro-RNAs. Different miRNAs are involved in gene expression, which are associated with differentiation, proliferation and neurogenesis of neuronal stem cells [13,70,71].

miRNAs	Gene Regulatory Mechanism(s)	Outcome(s)
miRNA-9	Interacts with Tlx	Controls NSC neurogenesis
Let-b	Interacts with Tlx and cyclinD	Represses NSC proliferation and increases differentiation
miRNA-124	Interacts with JAG1	Induces neural progenitor renewal
	Interacts with SOX9	Controls glial cell renewal
	Interacts with DLX2	Produces inter-neurons

Research on the DNA methylation status of PS1, PS2 and APP genes has yielded inconsistent results [72,73,74], with variable amounts of DNA methylation found in these genes, when compared to the severity of the disease. Some researchers have found no evidence of DNA methylation in the above-mentioned genes at all. However, evidence does exist that suggests that there are epigenetic dysregulations in the AD brain that may impact neurogenesis in any one of the ways described above. For example, there is a decreased amount of acetylation in histone 4 (H4K12Ac) that causes cognitive impairment, one of the major symptoms of AD [60]. Further, downregulation of miR-15, miR-16, miR-132 and miR-497 is also associated with the accumulation of phospho-tau, whereas miR-106a, miR-106b, miR-107, miR-124, miR-137, miR-153, miR-195 and miR-520c are linked to deposition of Aβ-plaques in AD [75,76,77]. Therefore, the expression profile of miRNAs can contribute significantly to the inhibition of neurogenesis, as well as the initiation of AD-like symptoms. Hence, upregulation of these miRNAs or identifying and restoring the status of the DNA-methylation might be a promising way to approach AD therapy.

## 5. Epigenetic Mechanisms in Induced Pluripotent Stem Cells

**Figure 4:**
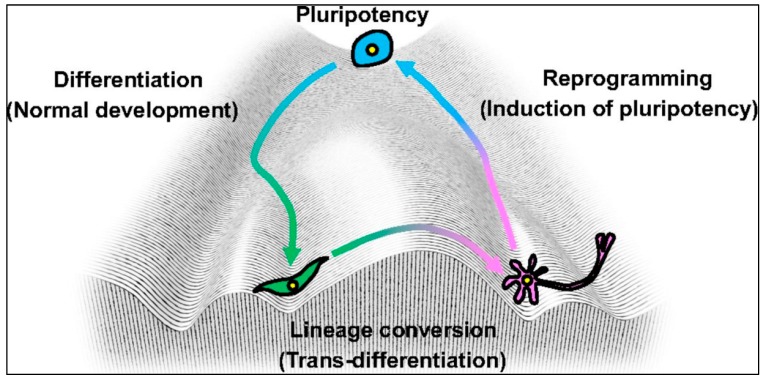
Cell reprogramming. Waddington’s epigenetic landscape showing the trans-differentiation of differentiated cells back to their pluripotent state, thus generating induced pluripotent stem cells (adopted from [78] Copyright 2013 with permission form Company of Biologists.

Epigenetics not only helps in stem cell proliferation and their maturation into specialized cells, but it also plays an important role in converting the already mature cell into another cell of a different lineage. Takahashi and colleagues [78] took advantage of this fact and modified Waddington’s landscape by inducing the already existing specialized cells into other somatic cell lineages, by the process of lineage conversion. In other words, the specialized cells moved from their determined path to another path on the landscape (Figure 4).

The reprogramming of cells into iPSCs or other somatic cell lineages by manipulating several genetic factors has been attempted by several scientists. However, the seminal pioneering work in this area was conducted by Shinya Yamanaka, who initially started with expressing 24 genes to induce the reprogramming process [79]. Through an exhaustive screening method to identify and isolate which genes play the critical role for this reprogramming process, Yamanaka and colleagues were able to focus their efforts on likely candidate genes, which included Oct3/4, Sox2, Klf4, c-Myc , Nanog and Lin-28 [80] (Table 5). Ultimately, out of the original 24 genes, Yamanaka and colleagues came to the conclusion that four of them (Oct4, Sox2, Klf4 and c-Myc) are necessary and sufficient to change a cell lineage. These factors are known as Yamanaka factors, and cells produced by this reprogramming method are known as induced pluripotent stem cells (iPSCs) [81].

**Table 5:** Role of different genetic factors involved in stem cell reprogramming. Summarizes the different genetic factors and their roles including the Yamanaka factors in reprogramming the stem cells into iPSCs.

Genes of Induction	Outcome(s) in the Presence of the Factor	YamanakA Factors	Outcome(s) in the Presence of the Factor
Sox family (Sox1, Sox2, Sox3, and Sox15)	Mainly associated with maintaining the pluripotency of the cell. Functions of Sox 2 are dosage dependent. Associated with early embryonic development (tissues and organ formation) [82].	Oct4	Associated with pluripotency and silenced when cells undergo differentiation [87].
Klf family (Klf1, Klf2, Klf4, and Klf5)	Associated with cell proliferation, differentiation and maintains tissue homeostasis and apoptosis [83].	Sox2	Associated with maintaining the embryonic stem cells in an undifferentiated state [82].
Myc family (c-myc, L-myc, and N-myc)	Associated with tumor or cancer formation [84].	Klf4	They are required for reprogramming and self-renewal of embryonic stem cells [83].
Nanog	Similar to Oct-3/4, they maintain pluripotency [85].	c-Myc	Associated with early reprogramming and cell proliferation. They are also associated in the process of the transcriptional activity of some of the genes that undergo de-differentiation and proliferation [15].
LIN28	Associated with maintaining pluripotency by regulating miR let 7 [86].		

Interestingly, it was found that when iPSCs are produced from embryonic stem cells (ESCs), the epigenetic profile and gene expression profile were maintained with minimal differences. However, the iPSCs derived from fibroblasts had about 3349 different methylated regions, but the iPSCs derived from the blood had only about 516 methylated regions [80]. Hence, these markers are initially not active, but become activated during reprogramming of the cells. In one study, DNA methylation status was compared between different ESCs, iPSCs and somatic cell lines, and it was found that about 90% of the regions had the same epigenetic status, while the remaining 10% were responsible for all of the differences between these cells [88]. Similar comparisons were made between MSCs, ESCs and iPSCs, and it was found that the methylation status was about 50% for MSCs and 70% for iPSCs that were derived from either MSCs or ESCs. Hence, reprogramming of the iPSCs from any of these cells requires changes of epigenetic status, namely converting the un-methylated regions to methylated regions [89].

Due to the variable expression of the methylation status, there is a shift in the way these iPSCs behave. For example, the iPSCs derived from mouse blood cells or the skin cells will have different methylation profiles, which influence how well these cells are reprogrammed to become hematopoietic or osteogenic lineages, respectively [90]. It has also been noted that decreased, or insufficient, DNA methylation affects the efficacy of the iPSCs that are derived from hepatocytes, fibroblasts and melanocytes [80]. As such, epigenetic memory, in the form of the methylation profile, plays an important role in reprogramming the cells and their characteristics. Some of the factors that have an influence on the epigenetic memory are the media used in the iPSC culture, the supplements and the nutrients (vitamin C and trichostatin, respectively), as well as the levels of O_2_ and CO_2_. Interestingly, unlike MSCs, the passage number does not appear to play a critical role in differentiation. The methylation status remains the same during higher and lower passages. The somatic cells from which iPSCs are derived have a noticeable impact on the epigenetic mechanism, whereas the number of passages does not [91]. *In vitro* reprogramming of these cells involves loss of repressive markers (H3K27me3) and the gain of activation markers (H3K4me3). Hence, when there is a transition from somatic cells back to pluripotent cells, another epigenetic marker, H3K4me2, is involved, which is lost in the somatic genes, but gained in the pluripotent cells. Similar to adult NSCs, iPSC reprogramming and maturation depends on epigenetic mechanisms and the efficiency by which cells transform into a different lineage. Manipulating the desired lineage for therapeutic purposes depends on controlling these specific epigenetic mechanisms. These also include mechanisms other than DNA methylation (for a review, see Paap and Plath, 2013) [92].

## 6. Conclusions

Understanding the epigenetic mechanisms influencing the differentiation of stem cells, in terms of passage number and culture conditions, including the use of appropriate supplements, are important variables for creating the type of cells that will provide the most effective treatment for neurodegenerative diseases. The use of MSCs, NSCs and iPSCs provides a promising tool for therapeutic treatments of such disorders. The “Holy Grail” for devising the most effective treatments for neurodegenerative diseases involves replacing lost neurons. Therefore, differentiation of MSCs, NSCs and iPSCs into neurons requires a thorough understanding of the epigenetic status of these cells at the time of their transplantation. Being able to manipulate these cells to a desired epigenetic status for transforming them into the appropriate neuronal lineages could provide the critical means for developing optimal cell therapies for neurodegenerative disorders.
